# Mr. Davies, on Medical Controversies

**Published:** 1807-09

**Authors:** H. Davies

**Affiliations:** Piccadilly


### *To the Editors of the Medical and Physical Journal.*

Gentlemen,

We were given to understand, that after an appeal to the Royal College of Physicians, for their opinion on Vaccination, private communications on that subject would be for the most part suspended; but as several pages of the last Number of your Journal were occupied in treading the beaten path of animadversions on the Anti-vaccinists, you will allow me to make an observation or two on medical controversies in general, and this in particular.

When new Theories in medicine are discovered, however ingenious in themselves, they will be received by experienced practitioners with caution, and examined with candor; nor should the discoverers be too sanguine in their expectations of a general adoption of their systems, till a sufficient period of time has elapsed to confirm the success of the practice.

It is possible that an opposite mode of treatment of the same disease may prove successful with patients of a different temperature. Hence the application of cold water, in burns and scalds as a powerful sedative, proves an appropriate remedy for a patient of a robust and vigorous constitution, when a more stimulant application, (such as the terebinthinate) is equally necessary to another, where inanition or the want of vital energy prevails. These circumstances considered, why should practitioners so furiously contend for the infallibility of the one, or the futility of the other treatment in the same case?

The same observation perhaps may be admitted respecting the cold ablution in gouty inflammation. It has been administered administered with success (doubtless) in one case, whilst a practitioner would be deterred, by a reverse of temperature in the patient, from adopting it in another; and as both the cold and warm treatment have both proved efficacious in this disease, (for facts are stubborn things) I humbly apprehend, the success has chiefly depended on the constitutional variety of their respective patients.

As to the Vaccine Contest, there has been hardly any cessation of arms since hostilities commenced ; and though I am no enemy to vaccination, believing it to be a general, though, perhaps, not an universal defence, against variolous infection; yet, I was sorry to find a zealous Correspondent in the last Number of the Journal raking the ashes of the dead, to support the almost thread-bare controversy. I think when the antagonist has quitted the field, that well known sentiment should be adopted, "*Nil, nisi bonum de mortuis.*" And as he has appeared before a higher tribunal, his hatchment should be allowed the accustomed motto, "*Requiescat in pace.*" Therefore, since the practice has been sanctioned by general approbation, throughout the greatest part of the habitable globe, it should seem needless to "*Ring*" another peal in the ears of the medical world on that long debated point.

It is, Sir, to be hoped, that your valuable miscellany will be furnished more with *practical* and less with *controversial** subjects in future. Your readers would be much better satisfied to find it a vehicle for useful information, than a field for endless contention; and as there is an ample scale for improvement in the "medical art," (for few will allow it has arrived to its "*Ne plus ultra,*" every correspondent should aim at the accomplishment of this grand and essential point.—Should these cursory hints have the least tendency to promote the purposes of general utility in a deservedly esteemed work, the writer will be amply gratified.

I am, &c.

H. DAVIES.

*Piccadilly, Aug.* 8, 1807.

---

* Mr. D. seems to forget, that without a stimulus all vital action is suspended. This stimulus, in Writers, is Controversy, or a discovery of new facts. The consequence is obvious. Ed.

